# Serum Amyloid A Protein as a Potential Biomarker for Severity and Acute Outcome in Traumatic Brain Injury

**DOI:** 10.1155/2019/5967816

**Published:** 2019-04-16

**Authors:** Evan Wicker, Leah Benton, Kershina George, William Furlow, Sonia Villapol

**Affiliations:** ^1^Department of Pharmacology & Physiology, Georgetown University, Washington, D.C., USA; ^2^M.S. Biochemistry and Molecular Biology Program, Georgetown University, Washington, D.C., USA; ^3^Center for Neuroregeneration and Department of Neurosurgery, Houston Methodist Research Institute, Houston, TX, USA

## Abstract

Traumatic brain injury (TBI) causes a wide variety of neuroinflammatory events. These neuroinflammatory events depend, to a greater extent, on the severity of the damage. Our previous studies have shown that the liver produces serum amyloid A (SAA) at high levels in the initial hours after controlled cortical impact (CCI) injury in mice. Clinical studies have reported detectable SAA in the plasma of brain injury patients, but it is not clear if SAA levels depend on TBI severity. To evaluate this question, we performed a mild to severe CCI injury in wild-type mice. We collected blood samples and brains at 1, 3, and 7 days after injury for protein detection by western blotting, enzyme-linked immunosorbent assay, or immunohistochemical analysis. Our results showed that severe CCI injury compared to mild CCI injury or sham mice caused an increased neuronal death, larger lesion volume, increased microglia/macrophage density, and augmented neutrophil infiltration. Furthermore, we found that the serum levels of SAA protein ascended in the blood in correlation with high neuroinflammatory and neurodegenerative responses. Altogether, these results suggest that serum SAA may be a novel neuroinflammation-based, and severity-dependent, biomarker for acute TBI.

## 1. Introduction

Traumatic brain injury (TBI) affects almost two million people in the US each year, leaving 2% of the population suffering from disabilities resulting from single, or multiple, brain injuries [1, 2]. In the phases that follow the initial impact, the secondary injury produces molecular and cellular changes that we can quantify [3, 4]. To date, TBI diagnosis relies on clinical judgment based on neurological examinations and brain imaging results. Currently, clinicians do not have a rapid and effective way to test the presence or severity of TBI. However, a quick diagnosis is critical to classify TBI according to its degree of severity to prevent long-term disability; biomarkers with high sensitivity and specificity are needed.

The local inflammation after TBI does not exclusively affect the brain; it also affects other organs, including the liver. Along with hepatic acute phase proteins (APP), such as serum amyloid P, C-reactive protein, complement proteins, and serum amyloid A (SAA) all influence the inflammation process [5]. Human SAA is a family of proteins consisting of SAA1, SAA2, and SAA4. SAA1 and SAA2 are the major APP primarily synthesized by hepatocytes; however, an extrahepatic production has been reported [6]. In mice, the SAA family is formed by three inducible genes, Saa1, Saa2, and Saa3, plus a constitutively expressed Saa4. SAA has low basal levels but high inducibility associated with its role in inflammation-associated neuropathologies [7, 8]. Specifically, up to 1000-fold increase levels of SAA were found in mice injected with lipopolysaccharides (LPS) [9]. Our laboratory has previously demonstrated that the SAA levels were increased in blood and liver at early hours after TBI in a mouse model [10].

Clinical studies have shown SAA concentrations in serum at day one after hypoxic-ischemic encephalopathy to be significantly correlated with the severity of damage in neonates [11]. Furthermore, SAA levels in adult TBI patients with severe polytrauma have also been reported [12]. However, it remains unknown if the circulating SAA levels depend explicitly on the severity of TBI. Precisely, there is a current lack of experimental models of TBI that interrogate SAA as a potential biomarker of injury severity.

In this study, we tested if SAA protein in serum could be a measure of the severity of the head injury. To do this, we used an experimental TBI model in mice, and we measured both the extent of the lesion and several cellular markers of the neuroinflammatory and neurodegenerative responses. Due to the need to find severity-dependent biomarkers of brain damage, SAA level quantification can be viewed as a sensitive and effective candidate to predict the severity of brain injury.

## 2. Materials and Methods

### 2.1. Mice and Controlled Cortical Impact (CCI) Injury Model

We used nine-week-old male C57BL/6J mice weighing 22-25 g. All mice were purchased from Jackson Laboratories and were kept under 12:12 hour light and dark cycle with access to food and water ad libitum. We anesthetized mice with isoflurane (3% for induction, 1.5% for maintenance) and performed controlled cortical impact (CCI) injury on the left side of the brain at the primary motor and somatosensory cortices using an electromagnetically driven CCI device (Impact One stereotaxic impactor; Leica Microsystems, Buffalo Grove, IL). The site of impact was located at 2 mm lateral and 2 mm posterior to the bregma, and the parameters used for injury severity correspond as follows: mild CCI (0.5 mm depth, 3.26 m/s) and severe CCI (2.5 mm depth, 5.25 m/s), with a 3 mm diameter flat impact tip. Sham mice received the same craniotomy without the impact injury. All animal studies were approved by the Georgetown University Institutional Animal Care and Use Committee (IACUC) and were conducted following the NRC Guide to the Care and Use of Laboratory Animals.

### 2.2. Tissue Extraction and Serum Collection

We sacrificed the animals at 1, 3, and 7 days post-injury (dpi) for the time course study, and at 1 day after injury for the injury severity study. All mice were euthanized with carbon dioxide (20%) and perfused with Phosphate-Buffered Saline (PBS). For serum collection, trunk blood was collected and allowed to clot for 10 minutes at room temperature, and serum was obtained by centrifugation (14,000 x g) at 4°C for 10 minutes and stored at -80°C. Brains were removed, postfixed in 4% paraformaldehyde overnight, and stored at 4°C in a 30% sucrose solution.

### 2.3. Western Blot Analysis for Detection of SAA

Serum samples were diluted 1:5 with Tris-Buffered Saline (TBS), combined with 2x Laemmli sample buffer (Bio-Rad), and heated at 95°C for 5 min before loading into 12% SDS-PAGE gel electrophoresis. Separated proteins were transferred onto nitrocellulose membranes. Following transfer, membranes were blocked in 5%* w/v* skim milk powder in TBS-Tween 20 (TSB-T) blocking buffer for 1 h at room temperature. Membranes were incubated with primary antibody in 5%* w/v* skim milk/TBS-T goat anti-SAA (1:500; AF2948, R&D Systems, Minneapolis, MN) overnight at 4°C. Following primary antibody incubation, membranes were washed for 3 x 5 min in TBS-T and probed with secondary horseradish peroxidase-conjugated rabbit anti-goat antibody (1:1000; Jackson ImmunoResearch Laboratories, West Grove, PA) in 3%* w/v *skim milk powder in TBS-T for 2 h at room temperature. Membranes were washed for 3 x 5 min in TBS-T and developed with SuperSignal West Pico enhanced chemiluminescence reagent (Thermo Scientific, Waltham, MA). An Amersham 600 imaging acquisition system (GE Healthcare, Chicago, IL) equipped with a cooled charge-coupled device camera was used for the detection of the proteins. After the detection of SAA protein, the membranes were blocked again with 5% casein-based blocking buffer for 30 minutes at room temperature and incubated overnight at 4°C with rabbit anti-glyceraldehyde-3-phosphate dehydrogenase (GAPDH) (1:1000; Invitrogen, Carlsbad, CA) acting as a loading control. After incubation, the membranes were washed, incubated with the anti-rabbit secondary antibody (1:1000; Jackson ImmunoResearch Laboratories, West Grove, PA) as before, and developed as above. Raw pixel intensities of bands from Western blots were quantified by densitometry using Image J software (NIH, Bethesda, MD).

### 2.4. ELISA Assay

A quantitative enzyme-linked immunosorbent assay (ELISA) (Aviva Systems) has been used to measure SAA1/2 in mouse serum (Aviva Systems, OKEH04394, San Diego, CA) according to the manufacturer's protocol. Absorbance was measured at 450 nm on a microplate reader purchased from Perkin Elmer (Waltham, MA).

### 2.5. Immunohistochemical Analysis

Serial microtome coronal brain sections (20 *μ*m thick) through the dorsal hippocampus were collected for immunohistochemistry analysis. Free-floating parallel brain sections were washed with PBS with 0.5% Triton X-100 (PBS-T) 3 x 5 min and blocked with 5% normal goat serum (NGS) in PBS-T for 1 h. Brain sections were incubated at 4°C overnight in PBS-T and 3% of NGS using the following primary antibodies: anti-rabbit Iba-1 antibody (1:500, Wako, Chemicals, Richmond, VA) for microglia/macrophages, and anti-rat Ly6B.2 alloantigen antibody (1:100, Bio-Rad, Hercules, CA) for neutrophils. After incubation, brain sections were washed in PBS-T and incubated with the corresponding anti-rabbit Alexa Fluor 568-conjugated or anti-rat Alexa Fluor 488-conjugated IgG secondary antibodies (all 1:1000, Invitrogen) for 2 h at room temperature. Sections were rinsed with PBS 3 x 5 min and incubated in PBS with DAPI solution (1:50,000, Sigma-Aldrich, St. Louis, MO) for counterstained nuclei. The sections were rinsed with distilled water and coverslipped with Fluoro-Gel with Tris Buffer mounting medium (Electron Microscopy Sciences, Hatfield, PA).

### 2.6. Histological Nissl Staining and Lesion Volume Measurements

Nissl staining was assessed on an average of 10 brain sections spaced equidistantly apart between 0 and -2.70 mm from bregma corresponding to the injured area. Brain slices were mounted on gelatin-coated glass slides (SuperFrost Plus, Thermo Fisher Scientific, IL) and stained for 10 min with 0.5% cresyl violet (Sigma-Aldrich, St. Louis, MO) dissolved in distilled water and filtered. Stained slides were dehydrated for 2 min using 100%, 95%, 70%, and 50% ethanol, cleared in xylene for 2 min (x2), covered with Permount mounting medium (Thermo Fisher Scientific) and coverslipped. All slides were scanned, and the lesion score ranged from 0 to 4; (0, no lesion; 1, small cortical lesion; 2 medium cortical lesion; 3, large cortical lesion and small hippocampal lesion; 4, large cortical and hippocampal lesion). The lesion score captures the depth and the speed of the impact into the cortex and how it increases the injury severity.

### 2.7. Cell Death Assay

Brain sections were processed for terminal deoxynucleotidyl transferase-mediated biotinylated-dUTP nick end labeling (TUNEL). Staining was performed by using the* in situ* Cell Death Detection Kit, Fluorescein (Roche, IL), according to the manufacturer's instructions. Coronal brain sections were mounted on gelatin-coated glass slides. Briefly, brain sections were rinsed with PBS and were incubated with kit-supplied TUNEL reaction mixture at 37°C for 1 h. For double staining, brain sections were incubated with primary antibody against monoclonal anti-mouse NeuN (1:200, Sigma-Aldrich) overnight at 4°C; Alexa Fluor 568-conjugated goat anti-mouse IgG (1:1,000, Invitrogen) were applied for 1 h at room temperature. Sections were rinsed with PBS 3 x 5 min and incubated in PBS with DAPI solution. The sections were rinsed with distilled water and coverslipped with Fluoro-Gel with Tris Buffer mounting medium. TUNEL-positive nuclei were counted in 5 cortical regions (x20) in the three to five coronal sections for each animal (n=5 mice/group at each time point).

### 2.8. Quantitative Analysis

For quantitative analysis of immunolabeled sections, we implemented unbiased standardized sampling techniques to measure tissue areas corresponding to the injured cortex showing positive immunoreactivity as we previously described [13, 14]. To quantify the number of TUNEL, NeuN, and Ly6B.2-positive cells, an average of four sections from the lesion epicenter (–1.34 to – 2.30 mm from bregma) were counted in cortical layers in 3 to 5 coronal sections for each animal, n = 5 mice per group, within each brain region, every positive cell in each of 5 cortical fields (x20, 151.894 mm^2^) around the impact area, as we have previously described [15]. For proportional area measurements, the microglial/macrophages (Iba-1 positive) activation was reported as the proportional area of tissue occupied by immunohistochemical stained cellular profiles within a defined target area. Thresholded images converted to 8-bit grayscale were made using ImageJ (NIH, Bethesda, MD). The thresholding function was then used to set a black and white threshold corresponding to the imaged field, with the averaged background subtracted out. Once a threshold is set, the “Analyze Particles” function was used to sum up the total area of positive staining and to calculate the fraction of the total area that is positive for the stain as previously was described [13, 14]. Data are shown as the percentage of Iba-1 positive immunoreactivity per the total area that occupied on the field studied. Images were acquired on an Axioplan 2 microscope (Zeiss, Thorwood, NY) with a Photometrics camera and analyzed using AxioVision software (4.8, SP1).

### 2.9. Statistical Analysis

All data in this study are expressed as the mean ± standard error of the mean (SEM). P < 0.05 or less was considered statistically significant. For data with a single time point after injury, intergroup differences were evaluated by one-way analysis of variance (ANOVA), followed by Dunnett's multiple comparison test when comparing all values to the control, and Bonferroni posttest when comparing selected values. For statistical comparison of mild and severe TBI data, the Mann-Whitney U test was used for groups of two. All statistics were performed with GraphPad Prism 5.0 software (GraphPad, San Diego, CA).

## 3. Results

### 3.1. SAA Expression in Blood Peaks at 1 Day after Injury and Is Severity Dependent

**Figure 1:**
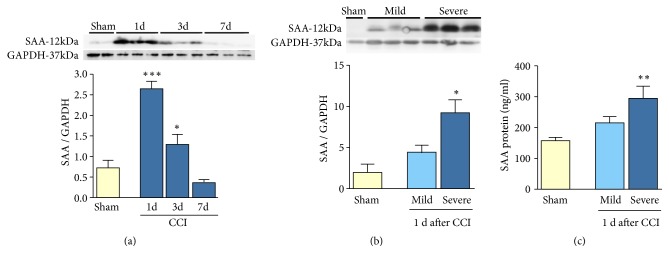
*SAA levels peak at 1 day after injury and increase with the injury severity*.* Time course of acute phase proteins in response to brain injury*. (a) SAA expression increases after injury in serum at 1 day after injury and returns to sham levels at 7 days after injury. At 1 day after CCI, severe injury has higher SAA expression than mild injury or sham mice as is shown by western blot (b) or by ELISA analysis (c). Protein expression in serum was normalized with GAPDH. Mean±sem, *∗*p<0.001, n=5/group.

We tested if SAA protein levels were acute or severity dependent. SAA protein levels were quantitated by western blot in blood serum collected in sham and injured mice at 1, 3, and 7 days after CCI (Figure 1(a)). Immunoblot analysis revealed a single band of molecular weight 12 kDa that corresponds to SAA1/SAA2, indicated as SAA. While SAA levels were slightly detectable in sham mice, in injured mice at 1 day after CCI, SAA levels reached their peak, increasing up to 3-fold change to sham animals. Subsequently, SAA levels decreased at 3 and 7 days after CCI with no significant difference observed in sham mice. Summarizing, TBI induced an acute increase in the levels of SAA in serum. SAA levels in serum were highest after severe CCI, lower after mild CCI, and nearly absent in sham mice (Figure 1(b)). To corroborate these results, we used a sensitive ELISA analysis and found an increase of 23% of SAA1/2 levels in the severe injury compared to mild CCI (Figure 1(c)). For these experiments, we used GAPDH protein as a control.

### 3.2. Injury Severity Increases Lesion Extension and Neuronal Cell Death

**Figure 2:**
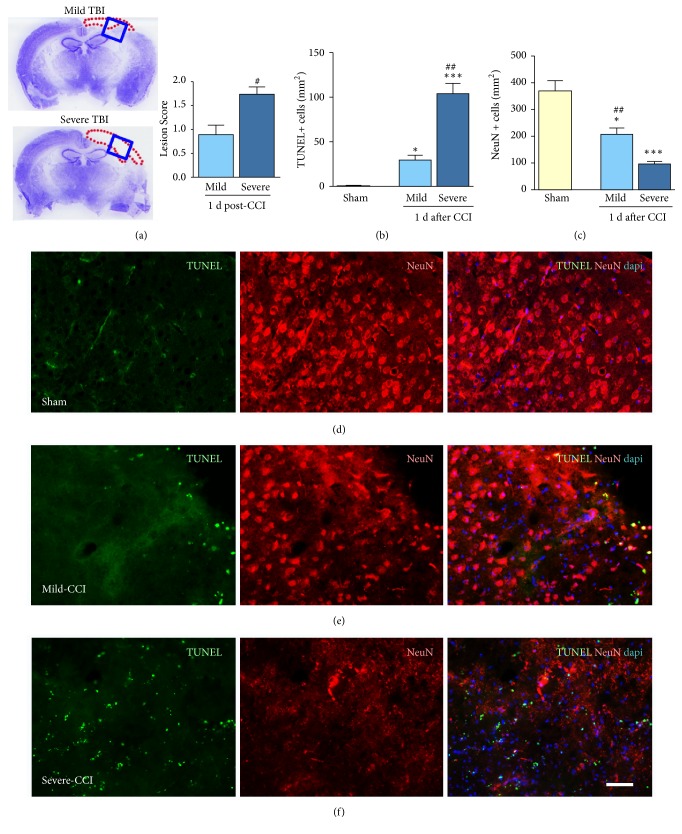
*Neuronal loss and cortical lesion are injury severity dependent. *Representative sections of injured brains stained with cresyl violet. Blue box represents the cortical brain region analyzed. Severe injury has an increased lesion score compared to mild injury at 1 day after CCI (a). The number of dying neurons around the cortical impact site in sham mice or after mild injury is fewer compared to severe injury (b). Increased numbers of NeuN positive cells in sham mice are reduced 2 folds after mild TBI, and 4 folds after severe injury (c). Representative images show the number of dying neurons around the cortical impact site in sham, and mild or severe injured mice indicated by TUNEL (green), NeuN (red), and DAPI positive cells (blue). Mean±sem, n=5/group, *∗∗∗*p<0.001, *∗∗*p<0.01, *∗*p<0.05 injured vs. sham, and #p<0.05 mild vs. severe injury.

We next examined SAA protein levels compared to lesion size. We observed larger lesions in mice that were severely injured compared to those that were mildly injured (Figure 2(a)). Next, we examined CCI-induced neuronal cell death in the pericontusional cortex and around regions to the impact site using TUNEL assay in combination with immunofluorescence staining for the neuronal marker, NeuN (Figures 2(d)–2(f)). We observed 71% more TUNEL positive cells in severely injured brains compared with mild brain injury (Figure 2(b)). At 1 day after injury, there was a 43% decrease in NeuN positive cells in mild CCI, and 75% in severe CCI compared with sham brains in the cortical regions (Figure 2(c)).

### 3.3. Injury Severity Increases Microglia/Macrophage Density and Neutrophil Infiltration into the Injured Brain

**Figure 3:**
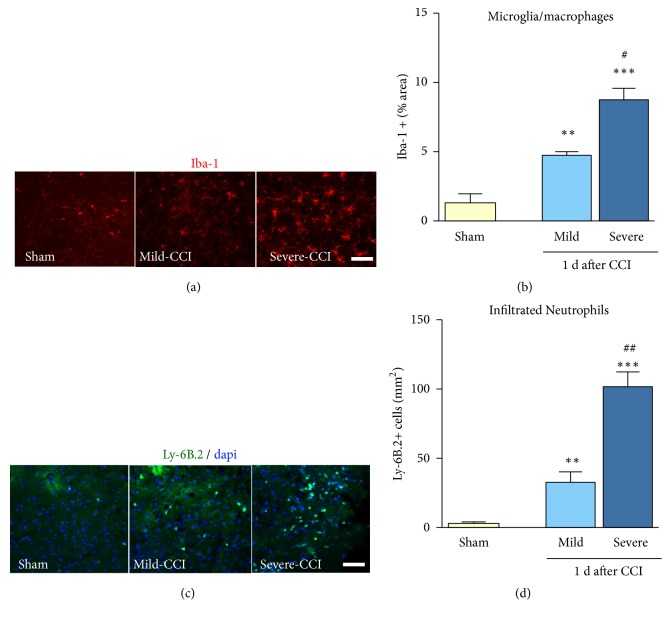
*Microglia activation and neutrophil infiltration are dependent on injury severity*. Immunofluorescence images show microglia/macrophages from sham to mild and severe injury (Iba-1, red) (a). Quantitative analysis of Iba-1 immunoreactivity is reduced in injured brains in sham mice and increased 3 folds after mild TBI and 5 folds after severe injury (b). Immunofluorescence images show infiltrated neutrophils from sham to mild and severe injury (Ly-6B.2, green; dapi, blue) (c). The number of Ly-6B.2 positive cells increased in injured brains 3 folds after mild TBI, and 5 folds after severe injury (d). Mean±sem, n=5/group, *∗∗∗*p<0.001, *∗∗*p<0.01 injured vs. sham, and #p<0.05, ##p<0.01 mild vs. severe injury.

Using immunohistochemistry, we detected Iba-1 positive cells and calculated the percentage of Iba-1 area in severe and mild injured brain cortical regions at 1 day after CCI. We observed that the total Iba-1 positive area was significantly higher in severely injured brains (80% increase in mild TBI vs. 30% in sham) (Figures 3(a) and 3(b)). CCI injury induces a rapid infiltration of neutrophils (Ly-6B.2) into the cortex. Sham-injured brains have little to no evidence of Ly-6B.2 positive cells in the cortex. At 1 day after CCI, we observed 18% more Ly-6B.2 positive cells in mild-CCI brains compared with sham and displayed a significant increase of 55% in the severe-CCI brains. Between mild and severe brain injury we observed a 3-fold increase in the number of infiltrated neutrophils (Figures 3(c) and 3(d)).

### 3.4. Increased SAA Protein Levels Correlate with Injury Severity

**Figure 4:**
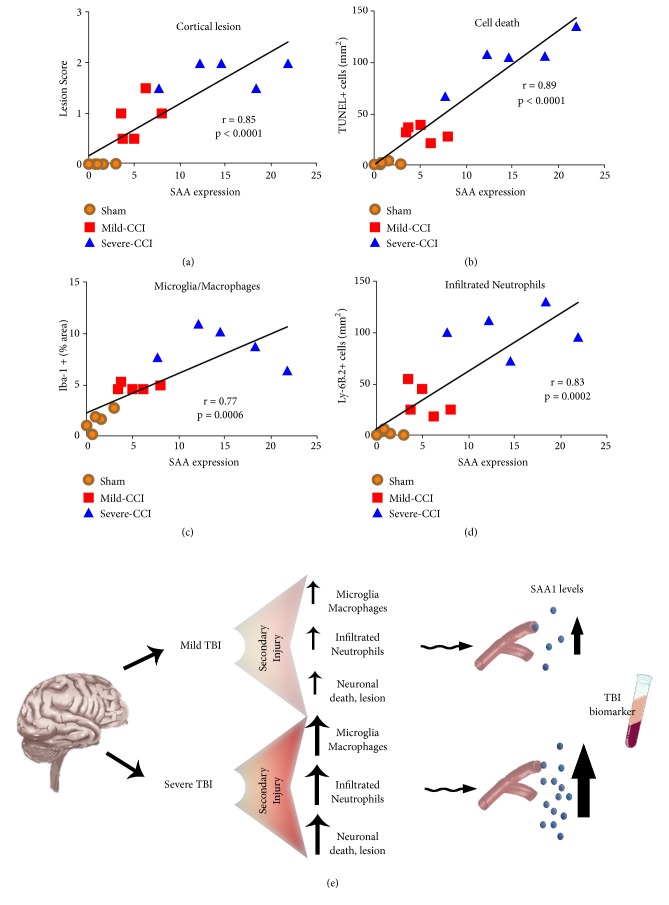
*SAA expression increases within injury severity.* Correlation between the SAA levels in serum after 1 day after injury with the lesion score (a), cell death (b), microglia/macrophages (c), and neutrophil infiltration (d) was assessed. (e) Summary of secondary injury processes that trigger neuropathological and inflammatory responses and release into the bloodstream. Mild to severe injury causes lower to higher SAA levels, respectively.

Finally, we examined SAA levels in correlation with lesion score, cell death, microglia/macrophages, and neutrophil infiltration. As illustrated in Figure 4(a), SAA in the blood is likely to reflect an injury-induced release of neurodegenerative features in the brain after 1 day after CCI. We found a positive correlation between SAA levels and lesion score (r = 0.85; p = 0.0001) and neuronal cell death (r = 0.89; p = 0.0001), (Figure 4(b)). SAA in the blood is likely to reflect an injury-induced release of neurodegenerative features in the brain after 1 day after CCI. We then evaluated the link between the SAA levels in serum and the microglia/macrophages and infiltrated neutrophils density. We found a positive correlation between SAA levels and microglia/macrophages density (r = 0.77; p = 0.0006) and neutrophil infiltration (r = 0.83; p = 0.0002), (Figures 4(c) and 4(d)).

## 4. Discussion

Here we found that increased brain injury severity, cell death, microglia/macrophages, and neutrophil infiltration correlate with increases in SAA protein. We have previously shown that SAA protein is produced at high levels by the liver in response to TBI in a mouse model [10]. Expression levels in liver proteins help to accurately and directly measure the severity of brain injury and should be a useful tool for TBI diagnosis when pathological symptoms or neuroimaging analysis do not provide this information. In this work, we have used a well-characterized experimental model of TBI to assess the severity of the lesion over time [14]. Our western blot analyses revealed that SAA was significantly overexpressed in the sera from TBI-mice compared to sham mice and was further validated by ELISA. We also found that SAA expression was higher in mice with severe TBI compared to mild TBI. The levels of SAA are of high sensitivity and severity dependent and thus may be a neuroinflammation-based diagnostic and prognostic biomarker for TBI patients (Figure 4(e)). While we did not differentiate between the SAA proteins, due to the low molecular weight of these proteins, it is difficult to find an antibody exclusive to one type of SAA.

The acute response of the TBI affects the systemic inflammatory state of the organs of the periphery [16]. The fastest form of communication between the brain and the periphery is via the release of extracellular vesicles to the circulation [17]. This peripheric inflammation mediated by cytokines and other small molecules promotes transmigration of leukocytes and an increase in systemic inflammation and damage in peripheral organs [17], including the liver. A recent study has shown how a single fluid percussion injury (FPI) episode in the rat cortex induces significant peripheral inflammatory and oxidative stress, disrupt redox statutes, and mitochondrial function in the liver [18]. High levels of circulating cytokines elicited by brain trauma affect the response injury in the brain and contribute to a blood-brain barrier opening and neutrophil infiltration [19]. Favorable changes to hepatic oxidative inflammation may exert protective effects on acute hyperglycemia and the cerebral inflammatory response induced by severe TBI. Furthermore, in our animal model of TBI, we observed that brain injury severity induced an increase in the infiltration of neutrophils in the brain lesion. A recent study demonstrated that the recruitment of leukocytes in the brain lesion occurs quickly and is maintained for at least a week [20]. The production of APP in the liver in response to a brain injury leads to the systemic inflammatory response syndrome [20], establishing a channel of communication between the liver and the extrahepatic organs, including the brain [21].

Hepatic SAA is increased in blood after TBI, which suggests that the SAA protein is transported rapidly in the circulation after brain trauma. Thus, SAA could be a potential TBI biomarker candidate but requires validation at the clinical level. Another compelling aspect of using SAA as a brain injury biomarker is its reliable detection at early time points [22]. Early detection is an essential property of SAA since early identification of intracranial injuries will assist clinical decisions and likely reduce long-term cognitive deficits, and potentially even death.

A first step for the majority of TBI patients is to perform a neuroimaging analysis to determine injury severity and localization. These tests, such as computerized tomography (CT) and magnetic resonance imaging (MRI) scans, are expensive and slow, especially concerning blood-based evaluations of TBI biomarkers. There currently exists a wide range of existing biomarkers for TBI in the blood, such as S100*β*, neurofilament heavy chain protein (NF-L), glial fibrillary acidic protein (GFAP), C-terminal ubiquitin hydrolase-1 (UCH-L1), neuron-specific enolase (NSE), myelin basic protein (MBP), and tau protein as well as other biomarkers detected in the cerebrospinal fluid (CSF) [23, 24]. Biomarkers in blood or CSF widely studied in TBI have been classified, together with the Glasgow coma scale (GCS), to provide additional information [25–28]. For example, serum levels of NF-L increase the first week after injury [29]. However, there are no* in vivo* studies that have fully assessed the serum half-life of NF-L. Another panel of three biomarkers, NSE, metallothionein 3, and neurogranin, were all identified in mild TBI patients during the first 6 hours after trauma [30]. Their levels in blood, however, were undetectable days after injury, and these markers require clinical validation.

Proteins produced in the brain after damage are detectable at extremely low concentrations in the plasma. Many of them suffer from proteolytic degradation in the blood, and their levels can be affected by the elimination of blood through the liver or kidneys [31]. All of these factors are critical for accurate diagnosis and for designing appropriate rehabilitation therapies. Recent studies have shown that healthy university athletes express serum biomarkers related to concussion in varying concentrations [31]. It is clear that serum biomarkers have high variability and, above all, demographic factors such as sex have not been considered.

Recently, a panel of high-sensitivity markers has been approved by the FDA to detect serum levels of GFAP and UCH-L1 within 12 hours after head injury. However, this TBI diagnostic has generated controversy as it does not measure the severity of the damage, nor does it diagnose a concussion, and the time of blood draw is critical to the measurement. There is a need to identify alternative serum biomarkers that may perform with respect to the limitations of current biomarkers. In summary, there remain multiple issues regarding the development of reliable TBI blood biomarkers.

Previous clinical studies have shown that SAA was elevated after TBI in humans [11, 12, 32–35], and the levels of SAA may be sensitive to injury severity. Clinical studies report that TBI patients with severe multitrauma showed higher serum SAA compared with mild to moderate only head trauma patients [12]. However, a correct association of SAA with brain trauma patients has not been established, mainly due to any validation in animal models. Therefore, we suggest that SAA may represent a sensitive biomarker that can be used to diagnose patients with mild to severe TBI within the first hours after injury, thus complementing the traditional GCS.

## 5. Conclusion

Collectively, our findings demonstrate that SAA production dynamics in serum appear to be correlated with injury severity, which increases or decreases in function of harmful secondary events, thus indicating SAA could act as a novel biomarker for monitoring TBI patients. Our work demonstrates, for the first time, in a mouse model of TBI that the SAA levels increase with the severity of the damage. SAA thus represents a promising new biomarker of the inflammatory and pathobiological responses to TBI severity. Although this needs to be further vetted and validated in clinically TBI patients, our findings reinforce the idea that TBI-related injuries are not limited to secondary damage in the brain. Additional studies are required to specifically block this response in the liver and thereby reduce neuroinflammation in the brain induced by the acute hepatic response.

## Data Availability

All data generated or analyzed to support the findings of this study are included within the article.
